# Supplementary material for: The role of ventromedial prefrontal cortex in reward valuation and future thinking during intertemporal choice
Source: eLife. 2021 Aug 3;10:e67387. doi: 10.7554/eLife.67387 (PMC8331177; doi:10.7554/eLife.67387)
Supplement: Source data 1. [file elife-67387-data1.doc]

**Main ANOVA on AREA UNDER THE CURVE (AUC)- Results section**

|  | SS | Degr. of Freedom | MS | F | p |
| --- | --- | --- | --- | --- | --- |
| Intercept | 24.75198 | 1 | 24.75198 | 224.9857 | 0.000000 |
| GROUP | 0.35367 | 1 | 0.35367 | 3.2148 | 0.078908 |
| Error | 5.61081 | 51 | 0.11002 |  |  |
| CONDITION | 1.75315 | 1 | 1.75315 | 54.3325 | 0.000000 |
| CONDITION*GROUP | 0.07036 | 1 | 0.07036 | 2.1805 | 0.145916 |
| Error | 1.64562 | 51 | 0.03227 |  |  |
| MAGNITUDE | 0.21451 | 1 | 0.21451 | 13.1701 | 0.000658 |
| MAGNITUDE*GROUP | 0.15460 | 1 | 0.15460 | 9.4918 | 0.003323 |
| Error | 0.83066 | 51 | 0.01629 |  |  |
| CONDITION*MAGNITUDE | 0.01380 | 1 | 0.01380 | 0.7556 | 0.388795 |
| CONDITION*MAGNITUDE*GROUP | 0.00246 | 1 | 0.00246 | 0.1345 | 0.715353 |
| Error | 0.93127 | 51 | 0.01826 |  |  |

Fisher Post-hoc comparison

|  |  |  |  |  |  |  |
| --- | --- | --- | --- | --- | --- | --- |
|  | GROUP | MAGNITUDE | {1} .38676 | {2} .52729 | {3} .35369 | {4} .36517 |
| 1 | HEALTHY | SMALL |  | 0.000000 | 0.669291 | 0.712443 |
| 2 | HEALTHY | LARGE | 0.000000 |  | 0.004076 | 0.040143 |
| 3 | VMPFC | SMALL | 0.669291 | 0.004076 |  | 0.756586 |
| 4 | VMPFC | LARGE | 0.712443 | 0.040143 | 0.756586 |  |

Cell statistics

(LEGENDA: TD = TEMPORAL DISCOUNTING IN THE STANDARD CONDITION; CD = TEMPORAL DISCOUNTING IN THE CUED CONDITION; SMALL = SMALL REWARD, LARGE = LARGE REWARD)

|  | Level of Factor | N | AUC TD SMALL Mean | AUC TD SMALL Std.Dev. | AUC TD SMALL Std.Err | AUC TD SMALL -95.00% | AUC TD SMALL +95.00% |
| --- | --- | --- | --- | --- | --- | --- | --- |
| Total |  | 53 | 0.270580 | 0.180671 | 0.024817 | 0.220781 | 0.320379 |
| GROUP | HEALTHY | 41 | 0.270053 | 0.190098 | 0.029688 | 0.210051 | 0.330056 |
| GROUP | VMPFC | 12 | 0.272381 | 0.151307 | 0.043679 | 0.176244 | 0.368517 |
|  |  |  | AUC TD LARGE Mean | AUC TD LARGE Std.Dev. | AUC TD LARGE Std.Err | AUC TD LARGE-95.00% | AUC TD LARGE +95.00% |
| Total |  |  | 0.358167 | 0.211817 | 0.029095 | 0.299783 | 0.416551 |
| GROUP | HEALTHY |  | 0.383176 | 0.224183 | 0.035012 | 0.312415 | 0.453937 |
| GROUP | VMPFC |  | 0.272718 | 0.137997 | 0.039836 | 0.185039 | 0.360397 |
|  |  |  | AUC CD SMALL Mean | AUC CD SMALL Std.Dev. | AUC CD SMALL Std.Err | AUC CD SMALL -95.00% | AUC CD SMALL +95.00% |
| Total |  |  | 0.487961 | 0.220273 | 0.030257 | 0.427246 | 0.548675 |
| GROUP | HEALTHY |  | 0.503463 | 0.215019 | 0.033580 | 0.435594 | 0.571331 |
| GROUP | VMPFC |  | 0.434995 | 0.239354 | 0.069096 | 0.282917 | 0.587073 |
|  |  |  | AUC CD LARGE Mean | AUC CD LARGE Std.Dev. | AUC CD LARGE Std.Err | AUC CD LARGE -95.00% | AUC CD LARGE +95.00% |
| Total |  |  | 0.622998 | 0.242025 | 0.033245 | 0.556287 | 0.689708 |
| GROUP | HEALTHY |  | 0.671401 | 0.233907 | 0.036530 | 0.597571 | 0.745231 |
| GROUP | VMPFC |  | 0.457620 | 0.198450 | 0.057288 | 0.331530 | 0.583709 |

**ANOVA on AUCs removing patients with lateral prefrontal damage – Results section**

|  |  |  |  |  |  |
| --- | --- | --- | --- | --- | --- |
|  | SS | Degr. of Freedom | MS | F | p |
| Intercept | 18.61354 | 1 | 18.61354 | 159.0902 | 0.000000 |
| GROUP | 0.17255 | 1 | 0.17255 | 1.4748 | 0.230652 |
| Error | 5.49900 | 47 | 0.11700 |  |  |
| CONDITION | 1.09076 | 1 | 1.09076 | 33.8444 | 0.000001 |
| CONDITION*GROUP | 0.09315 | 1 | 0.09315 | 2.8903 | 0.095722 |
| Error | 1.51474 | 47 | 0.03223 |  |  |
| MAGNITUDE | 0.07444 | 1 | 0.07444 | 4.8042 | 0.033380 |
| MAGNITUDE*GROUP | 0.20643 | 1 | 0.20643 | 13.3233 | 0.000657 |
| Error | 0.72821 | 47 | 0.01549 |  |  |
| CONDITION*MAGNITUDE | 0.00092 | 1 | 0.00092 | 0.0498 | 0.824316 |
| CONDITION*MAGNITUDE*GROUP | 0.01244 | 1 | 0.01244 | 0.6757 | 0.415215 |
| Error | 0.86523 | 47 | 0.01841 |  |  |

Fisher post hoc comparison

|  | GROUP | MAGNITUDE | {1} .38676 | {2} .52729 | {3} .39429 | {4} .35921 |
| --- | --- | --- | --- | --- | --- | --- |
| 1 | HEALTHY | SMALL |  | 0.000000 | 0.936168 | 0.696705 |
| 2 | HEALTHY | LARGE | 0.000000 |  | 0.063552 | 0.078606 |
| 3 | VMPFC | SMALL | 0.936168 | 0.063552 |  | 0.429401 |
| 4 | VMPFC | LARGE | 0.696705 | 0.078606 | 0.429401 |  |

Cell statistics

(LEGENDA:TD = TEMPORAL DISCOUNTING IN THE STANDARD CONDITION; CD = TEMPORAL DISCOUNTING IN THE CUED CONDITION; SMALL = SMALL REWARD, LARGE = LARGE REWARD)

|  | Level of Factor | N | AUC TD SMALL Mean | AUC TD SMALL Std.Dev. | AUC TD SMALL Std.Err | AUC TD SMALL -95.00% | AUC TD SMALL +95.00% |
| --- | --- | --- | --- | --- | --- | --- | --- |
| Total |  | 49 | 0.277393 | 0.185483 | 0.026498 | 0.224116 | 0.330670 |
| GROUP | HEALTHY | 41 | 0.270053 | 0.190098 | 0.029688 | 0.210051 | 0.330056 |
| GROUP | VMPFC | 8 | 0.315009 | 0.165771 | 0.058609 | 0.176422 | 0.453597 |
|  |  |  | AUC TD LARGE Mean | AUC TD LARGE Std.Dev. | AUC TD LARGE Std.Err | AUC TD LARGE-95.00% | AUC TD LARGE +95.00% |
| Total |  | 49 | 0.368883 | 0.213417 | 0.030488 | 0.307582 | 0.430183 |
| GROUP | HEALTHY | 41 | 0.383176 | 0.224183 | 0.035012 | 0.312415 | 0.453937 |
| GROUP | VMPFC | 8 | 0.295631 | 0.133431 | 0.047175 | 0.184079 | 0.407182 |
|  |  |  | AUC CD SMALL Mean | AUC CD SMALL Std.Dev. | AUC CD SMALL Std.Err | AUC CD SMALL -95.00% | AUC CD SMALL +95.00% |
| Total |  | 49 | 0.498581 | 0.217627 | 0.031090 | 0.436071 | 0.561090 |
| GROUP | HEALTHY | 41 | 0.503463 | 0.215019 | 0.033580 | 0.435594 | 0.571331 |
| GROUP | VMPFC | 8 | 0.473561 | 0.244373 | 0.086399 | 0.269260 | 0.677862 |
|  |  |  | AUC CD LARGE Mean | AUC CD LARGE Std.Dev. | AUC CD LARGE Std.Err | AUC CD LARGE -95.00% | AUC CD LARGE +95.00% |
| Total |  | 49 | 0.630810 | 0.249198 | 0.035600 | 0.559232 | 0.702388 |
| GROUP | HEALTHY | 41 | 0.671401 | 0.233907 | 0.036530 | 0.597571 | 0.745231 |
| GROUP | VMPFC | 8 | 0.422782 | 0.232541 | 0.082216 | 0.228372 | 0.617191 |

**ANOVA on AuCs with Testing site as a Factor – Supplementary Materials section**

|  | SS | Degr. of Freedom | MS | F | p |
| --- | --- | --- | --- | --- | --- |
| Intercept | 22.15029 | 1 | 22.15029 | 193.6362 | 0.000000 |
| TESTING SITE | 0.00539 | 1 | 0.00539 | 0.0472 | 0.828997 |
| GROUP | 0.28873 | 1 | 0.28873 | 2.5240 | 0.118556 |
| TESTING SITE *GROUP | 0.00076 | 1 | 0.00076 | 0.0066 | 0.935460 |
| Error | 5.60517 | 49 | 0.11439 |  |  |
| CONDITION | 1.50868 | 1 | 1.50868 | 45.8408 | 0.000000 |
| CONDITION* TESTING SITE | 0.01036 | 1 | 0.01036 | 0.3148 | 0.577331 |
| CONDITION*GROUP | 0.04747 | 1 | 0.04747 | 1.4424 | 0.235521 |
| CONDITION* TESTING SITE *GROUP | 0.00481 | 1 | 0.00481 | 0.1461 | 0.703923 |
| Error | 1.61265 | 49 | 0.03291 |  |  |
| MAGNITUDE | 0.21682 | 1 | 0.21682 | 12.9323 | 0.000749 |
| MAGNITUDE* TESTING SITE | 0.00022 | 1 | 0.00022 | 0.0132 | 0.909142 |
| MAGNITUDE*GROUP | 0.13371 | 1 | 0.13371 | 7.9749 | 0.006839 |
| MAGNITUDE* TESTING SITE *GROUP | 0.00756 | 1 | 0.00756 | 0.4509 | 0.505057 |
| Error | 0.82153 | 49 | 0.01677 |  |  |
| CONDITION*MAGNITUDE | 0.01321 | 1 | 0.01321 | 0.7450 | 0.392265 |
| CONDITION*MAGNITUDE* TESTING SITE | 0.03928 | 1 | 0.03928 | 2.2149 | 0.143090 |
| CONDITION*MAGNITUDE*GROUP | 0.01234 | 1 | 0.01234 | 0.6959 | 0.408202 |
| CONDITION*MAGNITUDE* TESTING SITE *GROUP | 0.00030 | 1 | 0.00030 | 0.0171 | 0.896477 |
| Error | 0.86894 | 49 | 0.01773 |  |  |

Fisher post hoc comparison

|  | GROUP | MAGNITUDE | {1} .38676 | {2} .52729 | {3} .35369 | {4} .36517 |
| --- | --- | --- | --- | --- | --- | --- |
| 1 | HEALTHY | SMALL |  | 0.000000 | 0.675371 | 0.717627 |
| 2 | HEALTHY | LARGE | 0.000000 |  | 0.004836 | 0.044198 |
| 3 | VMPFC | SMALL | 0.675371 | 0.004836 |  | 0.760024 |
| 4 | VMPFC | LARGE | 0.717627 | 0.044198 | 0.760024 |  |

Cell statistics (Testing s. = TESTING SITE, TD = TEMPORAL DISCOUNTING IN THE STANDARD CONDITION; CD = TEMPORAL DISCOUNTING IN THE CUED CONDITION; SMALL = SMALL REWARD, LARGE = LARGE REWARD)

|  | Level of Factor | Level of Factor | N | AUC TD SMALL Mean | AUC TD SMALL Std.Dev. | AUC TD SMALL Std.Err | AUC TD SMALL -95.00% | AUC TD SMALL +95.00% |
| --- | --- | --- | --- | --- | --- | --- | --- | --- |
| Total | TESTING S. | GROUP | 53 | 0.270580 | 0.180671 | 0.024817 | 0.220781 | 0.320379 |
| TESTING S. *GROUP | ITALY | HEALTHY | 27 | 0.254103 | 0.200719 | 0.038628 | 0.174701 | 0.333505 |
| TESTING S. *GROUP | ITALY | VMPFC | 4 | 0.247577 | 0.088690 | 0.044345 | 0.106451 | 0.388703 |
| TESTING S. *GROUP | CANADA | HEALTHY | 14 | 0.300815 | 0.170495 | 0.045567 | 0.202374 | 0.399256 |
| TESTING S. *GROUP | CANADA | VMPFC | 8 | 0.284782 | 0.179103 | 0.063322 | 0.135049 | 0.434516 |
|  |  |  |  | AUC TD LARGE Mean | AUC TD LARGE Std.Dev. | AUC TD LARGE Std.Err | AUC TD LARGE-95.00% | AUC TD LARGE +95.00% |
| Total |  |  |  | 0.358167 | 0.211817 | 0.029095 | 0.299783 | 0.416551 |
| TESTING S. *GROUP | ITALY | HEALTHY |  | 0.384262 | 0.231755 | 0.044601 | 0.292583 | 0.475941 |
| TESTING S. *GROUP | ITALY | VMPFC |  | 0.313407 | 0.125948 | 0.062974 | 0.112996 | 0.513818 |
| TESTING S. *GROUP | CANADA | HEALTHY |  | 0.381081 | 0.217284 | 0.058072 | 0.255626 | 0.506537 |
| TESTING S. *GROUP | CANADA | VMPFC |  | 0.252374 | 0.147335 | 0.052091 | 0.129199 | 0.375548 |
|  |  |  |  | AUC CD SMALL Mean | AUC CD SMALL Std.Dev. | AUC CD SMALL Std.Err | AUC CD SMALL -95.00% | AUC CD SMALL +95.00% |
| Total |  |  |  | 0.487961 | 0.220273 | 0.030257 | 0.427246 | 0.548675 |
| TESTING S. *GROUP | ITALY | HEALTHY |  | 0.533422 | 0.233233 | 0.044886 | 0.441158 | 0.625686 |
| TESTING S. *GROUP | ITALY | VMPFC |  | 0.459606 | 0.294214 | 0.147107 | -0.008555 | 0.927767 |
| TESTING S. *GROUP | CANADA | HEALTHY |  | 0.445684 | 0.167337 | 0.044723 | 0.349066 | 0.542302 |
| TESTING S. *GROUP | CANADA | VMPFC |  | 0.422689 | 0.228933 | 0.080940 | 0.231296 | 0.614082 |
|  |  |  |  | AUC CD LARGE Mean | AUC CD LARGE Std.Dev. | AUC CD LARGE Std.Err | AUC CD LARGE -95.00% | AUC CD LARGE +95.00% |
| Total |  |  |  | 0.622998 | 0.242025 | 0.033245 | 0.556287 | 0.689708 |
| TESTING S. *GROUP | ITALY | HEALTHY |  | 0.667207 | 0.214171 | 0.041217 | 0.582483 | 0.751930 |
| TESTING S. *GROUP | ITALY | VMPFC |  | 0.463928 | 0.284999 | 0.142500 | 0.010431 | 0.917425 |
| TESTING S. *GROUP | CANADA | HEALTHY |  | 0.679489 | 0.276587 | 0.073921 | 0.519793 | 0.839186 |
| TESTING S. *GROUP | CANADA | VMPFC |  | 0.454466 | 0.164445 | 0.058140 | 0.316986 | 0.591945 |
|  |  |  |  |  |  |  |  |  |
|  |  |  |  |  |  |  |  |  |

**ANOVA on the number of Inconsistent Choices – Results Section**

|  | SS | Degr. of Freedom | MS | F | p |
| --- | --- | --- | --- | --- | --- |
| Intercept | 108.8181 | 1 | 108.8181 | 170.9451 | 0.000000 |
| GROUP | 0.9879 | 1 | 0.9879 | 1.5519 | 0.218547 |
| Error | 32.4649 | 51 | 0.6366 |  |  |
| CONDITION | 0.0194 | 1 | 0.0194 | 0.0211 | 0.885008 |
| CONDITION*GROUP | 4.6043 | 1 | 4.6043 | 5.0103 | 0.029590 |
| Error | 46.8674 | 51 | 0.9190 |  |  |
| MAGNITUDE | 0.2424 | 1 | 0.2424 | 0.7845 | 0.379937 |
| MAGNITUDE*GROUP | 0.0726 | 1 | 0.0726 | 0.2349 | 0.630015 |
| Error | 15.7576 | 51 | 0.3090 |  |  |
| CONDITION*MAGNITUDE | 0.0902 | 1 | 0.0902 | 0.2192 | 0.641623 |
| CONDITION*MAGNITUDE*GROUP | 0.9393 | 1 | 0.9393 | 2.2827 | 0.136998 |
| Error | 20.9853 | 51 | 0.4115 |  |  |

Fisher post hoc comparison

|  | GROUP | CONDITION | {1} .93902 | {2} .60976 | {3} .75000 | {4} 1.1250 |
| --- | --- | --- | --- | --- | --- | --- |
| 1 | HEALTHY | STANDARD |  | 0.032413 | 0.312151 | 0.365754 |
| 2 | HEALTHY | EFT | 0.032413 |  | 0.494822 | 0.007542 |
| 3 | VMPFC | STANDARD | 0.312151 | 0.494822 |  | 0.181357 |
| 4 | VMPFC | EFT | 0.365754 | 0.007542 | 0.181357 |  |

Cell statistics

(LEGENDA: IC = INCONSISTENT CHOICES, TD = TEMPORAL DISCOUNTING IN THE STANDARD CONDITION; CD = TEMPORAL DISCOUNTING IN THE CUED CONDITION; SMALL = SMALL REWARD, LARGE = LARGE REWARD)

|  | Level of Factor | N | IC TD SMALL Mean | IC TD SMALL Std.Dev. | IC TD SMALL Std.Err | IC TD SMALL -95.00% | IC TD SMALL +95.00% |
| --- | --- | --- | --- | --- | --- | --- | --- |
| Total |  | 53 | 0.905660 | 0.766213 | 0.105247 | 0.694466 | 1.116855 |
| GROUP | HEALTHY | 41 | 0.902439 | 0.830809 | 0.129751 | 0.640203 | 1.164675 |
| GROUP | VMPFC | 12 | 0.916667 | 0.514929 | 0.148647 | 0.589497 | 1.243837 |
|  |  |  | IC TD LARGE Mean | IC TD LARGE Std.Dev. | IC TD LARGE Std.Err | IC TD LARGE-95.00% | IC TD LARGE +95.00% |
| Total |  | 53 | 0.886792 | 0.750907 | 0.103145 | 0.679817 | 1.093768 |
| GROUP | HEALTHY | 41 | 0.975610 | 0.790184 | 0.123406 | 0.726197 | 1.225022 |
| GROUP | VMPFC | 12 | 0.583333 | 0.514929 | 0.148647 | 0.256163 | 0.910503 |
|  |  |  | IC CD SMALL Mean | IC CD SMALL Std.Dev. | IC CD SMALL Std.Err | IC CD SMALL -95.00% | IC CD SMALL +95.00% |
| Total |  | 53 | 0.773585 | 0.775627 | 0.106540 | 0.559796 | 0.987374 |
| GROUP | HEALTHY | 41 | 0.682927 | 0.722462 | 0.112830 | 0.454890 | 0.910964 |
| GROUP | VMPFC | 12 | 1.083333 | 0.900337 | 0.259905 | 0.511287 | 1.655380 |
|  |  |  | IC CD LARGE Mean | IC CD LARGE Std.Dev. | IC CD LARGE Std.Err | IC CD LARGE -95.00% | IC CD LARGE +95.00% |
| Total |  | 53 | 0.679245 | 0.778894 | 0.106989 | 0.464555 | 0.893935 |
| GROUP | HEALTHY | 41 | 0.536585 | 0.674446 | 0.105331 | 0.323704 | 0.749467 |
| GROUP | VMPFC | 12 | 1.166667 | 0.937437 | 0.270615 | 0.571048 | 1.762286 |

**ANOVA on R^2^ – Materials and methods section**

|  | SS | Degr. of Freedom | MS | F | p |
| --- | --- | --- | --- | --- | --- |
| Intercept | 50.08448 | 1 | 50.08448 | 244.2193 | 0.000000 |
| GROUP | 0.04251 | 1 | 0.04251 | 0.2073 | 0.650854 |
| Error | 10.45908 | 51 | 0.20508 |  |  |
| CONDITION | 1.15315 | 1 | 1.15315 | 7.2018 | 0.009796 |
| CONDITION*GROUP | 0.64001 | 1 | 0.64001 | 3.9971 | 0.050921 |
| Error | 8.16608 | 51 | 0.16012 |  |  |
| MAGNITUDE | 0.00551 | 1 | 0.00551 | 0.1019 | 0.750867 |
| MAGNITUDE*GROUP | 0.15905 | 1 | 0.15905 | 2.9403 | 0.092468 |
| Error | 2.75881 | 51 | 0.05409 |  |  |
| CONDITION*MAGNITUDE | 0.07409 | 1 | 0.07409 | 1.2022 | 0.278024 |
| CONDITION*MAGNITUDE*GROUP | 0.04224 | 1 | 0.04224 | 0.6855 | 0.411559 |
| Error | 3.14297 | 51 | 0.06163 |  |  |

Fisher post hoc comparison

|  | GROUP | COND | {1} .62008 | {2} .57514 | {3} .71753 | {4} .41002 |
| --- | --- | --- | --- | --- | --- | --- |
| 1 | HEALTHY | STANDARD |  | 0.475354 | 0.358172 | 0.036632 |
| 2 | HEALTHY | EFT | 0.475354 |  | 0.154165 | 0.122350 |
| 3 | VMPFC | STANDARD | 0.358172 | 0.154165 |  | 0.010360 |
| 4 | VMPFC | EFT | 0.036632 | 0.122350 | 0.010360 |  |

Cell statistics

(LEGENDA: TD = TEMPORAL DISCOUNTING IN THE STANDARD CONDITION; CD = TEMPORAL DISCOUNTING IN THE CUED CONDITION; SMALL = SMALL REWARD, LARGE = LARGE REWARD)

|  | Level of Factor | N | R^2^ TD SMALL Mean | R^2^ TD SMALL Std.Dev. | R^2^ TD SMALL Std.Err | R^2^ TD SMALL -95.00% | R^2^ TD SMALL +95.00% |
| --- | --- | --- | --- | --- | --- | --- | --- |
| Total |  | 53 | 0.653035 | 0.301815 | 0.041458 | 0.569845 | 0.736226 |
| GROUP | HEALTHY | 41 | 0.653426 | 0.325421 | 0.050822 | 0.550711 | 0.756142 |
| GROUP | VMPFC | 12 | 0.651701 | 0.213379 | 0.061597 | 0.516127 | 0.787275 |
|  |  |  | R^2^ TD LARGE Mean | R^2^ TD LARGE Std.Dev. | R^2^ TD LARGE Std.Err | R^2^ TD LARGE-95.00% | R^2^ TD LARGE +95.00% |
| Total |  | 53 | 0.631254 | 0.335067 | 0.046025 | 0.538898 | 0.723610 |
| GROUP | HEALTHY | 41 | 0.586734 | 0.360837 | 0.056353 | 0.472840 | 0.700628 |
| GROUP | VMPFC | 12 | 0.783364 | 0.156960 | 0.045311 | 0.683636 | 0.883091 |
|  |  |  | R^2^ CD SMALL Mean | R^2^ CD SMALL Std.Dev. | R^2^ CD SMALL Std.Err | R^2^ CD SMALL -95.00% | R^2^ CD SMALL +95.00% |
| Total |  | 53 | 0.574859 | 0.346975 | 0.047661 | 0.479221 | 0.670497 |
| GROUP | HEALTHY | 41 | 0.619426 | 0.324522 | 0.050682 | 0.516994 | 0.721858 |
| GROUP | VMPFC | 12 | 0.422589 | 0.391749 | 0.113088 | 0.173683 | 0.671494 |
|  |  |  | R^2^ CD LARGE Mean | R^2^ CD LARGE Std.Dev. | R^2^ CD LARGE Std.Err | R^2^ CD LARGE -95.00% | R^2^ CD LARGE +95.00% |
| Total |  | 53 | 0.500651 | 0.406096 | 0.055782 | 0.388717 | 0.612585 |
| GROUP | HEALTHY | 41 | 0.530855 | 0.408842 | 0.063850 | 0.401809 | 0.659902 |
| GROUP | VMPFC | 12 | 0.397454 | 0.395916 | 0.114291 | 0.145901 | 0.649007 |
